# Seasonal Variation of Gut Microbial Composition and Metabolism in *Tibetan antelopes* in Hoh Xil National Nature Reserve

**DOI:** 10.3390/ani13223569

**Published:** 2023-11-18

**Authors:** Hang Zhao, Feng Jiang, Haifeng Gu, Hongmei Gao, Jingjie Zhang, Meng Zhang, Tongzuo Zhang

**Affiliations:** 1 Key Laboratory of Adaptation and Evolution of Plateau Biota, Northwest Institute of Plateau Biology, Chinese Academy of Sciences, Xining 810001, China; zhaohang21@mails.ucas.ac.cn (H.Z.);; 2 College of Life Sciences, University of Chinese Academy of Sciences, Beijing 100049, China; 3 Qinghai Provincial Key Laboratory of Animal Ecological Genomics, Xining 810001, China

**Keywords:** *Tibetan antelope*, 16S rRNA gene sequencing, untargeted metabolomics, seasonal variation, metabolic functions, metabolic pathways

## Abstract

**Simple Summary:**

The gut microbiota and metabolites play a crucial role in the physiological health of the host and are influenced by various factors, including seasons. We utilized 16s sequencing and untargeted metabolomics to uncover the impact of seasonal change on the gut microbiota and metabolites of *Tibetan antelopes*. Overall, this study revealed differences in the gut microbiota and metabolites of *Tibetan antelopes* during the cold and warm seasons. These findings can provide a basis for the conservation of *Tibetan antelopes*.

**Abstract:**

The Tibetan antelope is an endangered species suffering from poaching and habitat fragmentation. The intestinal flora and metabolites play a crucial role in the physiological homeostasis of hosts, which are influenced by various environmental factors like seasonal variation. In this particular research, our main goal was to explore the alterations in the metabolism and gut microbiota of *Tibetan antelopes* between the cold season (XB) and warm season (DA), using untargeted metabolomics and 16S rRNA gene-sequencing analyses. The findings indicated that *Tibetan antelopes* had a higher alpha-diversity of intestinal microbes during the cold season than during the warm season. Principal co-ordinate analysis revealed notable seasonal discrepancies in the function and structure of intestinal microbes in *Tibetan antelopes*. The relative abundance of *Firmicutes* was significantly increased during the cold season compared to during the warm season. Furthermore, the Tibetan antelope’s primary metabolic functions of the intestinal micro-organisms were significantly higher during the cold season. The untargeted metabolomics analysis results showed a total of 532 metabolites that were significantly different between the cold season and warm season groups. These metabolites were found to be enriched in a total of 62 metabolic pathways. Among the most significant pathways of enrichment were the purine metabolism and pyrimidine metabolism. The levels of related metabolites in those pathways were remarkably higher in the warm season compared to the cold season. The comprehensive analysis of 16S rRNA and the metabolome reveals there is a significant correlation between differential microbiota and differential metabolites. Therefore, the gut microbiota changes caused by seasonal changes influenced the metabolites as well. This research reveals the function of seasonal changes in the intestinal flora and metabolites in the adaptation of *Tibetan antelopes* to environmental fluctuations and supplies a theoretical basis for instructing the protection management of *Tibetan antelopes*.

## 1. Introduction

The Tibetan antelope (*Pantholops hodgsonii*), is a species that is unique to the Tibetan Plateau. It mainly inhabits alpine grasslands between 3700 and 5500 m above the sea level. The *Tibetan antelopes* were previously widely distributed in the Tibetan Plateau region, with a population of millions. However, due to the increasing human population and the rapid growth of livestock farming in the Tibetan Plateau, the distribution range of *Tibetan antelopes* has been further compressed to areas with a sparse population, high altitude, and poor conditions. The valuable fur of *Tibetan antelopes* has made them targets of rampant poaching, leading to a rapid decline in their population. By 1995, the total number of *Tibetan antelopes* in China had sharply dropped to about 50,000 to 75,000 individuals. The region where *Tibetan antelopes* are distributed is rich in mineral resources. However, gold panning and mining activities have severely damaged the habitat of *Tibetan antelopes*, leading to a reduction in the habitat area and gradual fragmentation. At the same time, with the construction of infrastructure, roads and railways have further extended into the habitat of *Tibetan antelopes*, causing destruction and disturbance to their survival. These factors have had an impact on the survival and normal migration of *Tibetan antelopes*, pushing them to the brink of extinction. Therefore, *Tibetan antelopes* are listed as a first-class nationally protected animal, strictly prohibiting and restricting all *Tibetan antelope* products.

Furthermore, the intestinal micro-organism is a sophisticated product of a lengthy period of evolution between the parasitifer and microbiota [1], and it is essential within the host’s body [2]. Healthy gut microbiota help with substance metabolism, immune regulation, nutrient digestion and absorption, and defending against pathogens [3,4]. Many environmental factors, for example, temperature, diet, and habitat, influence the structure and function of intestinal flora [5,6,7,8,9]. For instance, seasonal variations in the constitution of food alter the function, abundance, and composition of the intestinal micro-organisms in a number of animals [10,11]. Metabolites reflect continuous life activities over a given period of time and the impact of changes in the environment, physiological conditions, and pathological conditions on the body [12]. Analyzing changes in the metabolite content and understanding the relationship between metabolites and gut microbiota can provide insights into how the environment affects gut microbiota and how hosts adapt to environmental changes [13].

In this research, we utilized high-throughput 16S rRNA sequencing and untargeted metabolomics to conduct a comprehensive analysis of the variations in metabolites, microbial species, and functions between the cold and warm seasons in *Tibetan antelopes*. This research aimed to explore (i) the differences in the composition, abundance, and function of intestinal micro-organisms between the cold and warm season in *Tibetan antelopes*; and (ii) the metabolic characteristics in the intestines of *Tibetan antelopes* and their seasonal differences. This study establishes a foundation for understanding the metabolic function and gut microbiota diversity of *Tibetan antelopes*, along with providing a theoretical foundation for developing more effective protection methods for *Tibetan antelopes*.

## 2. Materials and Methods

### 2.1. Study Object and Sample Collection

In March and July of 2023, we collected fresh fecal samples from *Tibetan antelopes* within the Hoh Xil National Nature Reserve in Qinghai Province, China. We obtained 17 samples during the warm season and 23 samples during the cold season. After observing the defecation behavior of the *Tibetan antelopes* in the field, we recorded detailed information about the sampling locations and waited for them to leave in order to collect the fresh stool samples. The collected samples were temporarily stored in liquid nitrogen and then relocated to an ultra-low-temperature freezer set at −80 °C for preservation.

### 2.2. 16S rRNA Analysis

#### 2.2.1. DNA Extraction and Sequencing

Following the manufacturer’s instructions, we used the MinkaGene Stool DNA Kit (Microchip) to extract DNA from all of the samples. The concentration and purity of the extracted DNA were monitored by the NanoDrop One (Thermo Scientific, Waltham, MA, USA), and we performed electrophoresis on 1% Agarose gel to confirm the DNA’s quality. The PCR primer sets 515F (5′-GTGYCAGCMGCCGCGGTAA-3′) and 806R (5′-GGACTACHVGGGTWTCTAAT-3′) were used to amplify the hypervariable V4 region of the 16S bacterial rRNA gene [14]. Sequencing libraries were created using the NEBNext^®^ Ultra™ DNA Library Prep Kit for Illumina^®^ according to the manufacturer’s instructions. The sequencing was performed on an Illumina NoveSeq 6000 platform using the 2  ×  250PE rapid run mode.

#### 2.2.2. Bioinformatics and Statistical Analyses

The bioinformatics of microbes was operated using QIIME 2 2022.02 [15]. The raw sequence data were subjected to a quality filtering process using the q2-demux plugin. After that, the data were denoised using the DADA2 algorithm [16]. In the DADA2 pipeline, reads are cut to 220-based before being processed with the dada2 denoise pairing method. All amplicon sequence variants (ASV) are aligned with mafft [17] and are used to structure phylogenetic relationships with fasttree2 [18]. The α diversity index at the ASV level, for instance, the Shannon Diversity Index and the Observed Species Diversity Index, was calculated using the QIIME2 ASV table; then, we visualized these indices as a box plot (R software, package “ggplot2” v3.2.0) [19,20]. The β diversity analysis method was used to study the structural differences among the microbial communities in the various samples using UniFrac distance measurements, which were either weighted or unweighted, and they were visualized via principal co-ordinate analysis (PCoA) (R software, the “ape” package) [21]. The study employed the linear discriminant analysis effect size (LEfSe) to identify taxa with significant differences in abundance between groups at various levels. To determine statistical significance, an LDA score greater than 1 and a p-value less than 0.05 were used. The taxonomy was assigned to ASV using the q2-feature-classifier [22] for the naive Bayes classifier Sklearn taxonomy classifier versus the SILVA SSU Ref NR 99 database (version 138.1) reference sequences [23]. We provided a clear classification of bacteria, in terms of both their phylum and genus. We utilized PICRUSt2 software (v2.3.0) to determine the relative abundance of the predicted gene families in conjunction with the Kyoto Encyclopedia of Genes and Genomes (KEGG) database [24]. After considering the suggestion from the authors of PICRUSt2, we decided to exclude ASVs that had a weighted nearest sequence taxon index (NSTI) value greater than 2. This was performed because these ASVs had a low quality of prediction.

### 2.3. Metabolomic Analysis

#### 2.3.1. Metabolite Extraction

We took fecal samples and ground them using liquid nitrogen. Then, we mixed the homogenates with 80% methanol by vortexing them well. After that, we placed the sample in ice for 5 min and then centrifuged them for 20 min at 15,000× *g* and 4 °C. Some of the supernatants were diluted with LC-MS-grade water to a final concentration containing 53% methanol. These samples were then transferred to fresh Eppendorf tubes and centrifuged again for 20 min at 15,000× *g* and 4 °C. Finally, we injected the supernatant into the LC-MS/MS system for analysis [25].

#### 2.3.2. LC–MS/MS

UHPLC-MS/MS analyses were carried out using Vanquish UHPLC systems (ThermoFisher, Hennigsdorf, Germany) and Optirap Q ExactiveTM HF Mass Spectrometers (Orbitrap Q ExactiveTM HF-X Mass Spectrometers from Novogene Co., Ltd., Guangzhou, China). The experiment involved injecting samples onto a Hypersil Gold column. The injection was performed using a linear gradient over a period of 17 min, with a flow rate of 0.2 mL/min. The elution solvent used in positive polarity mode consisted of two components, eluent A, which was a 0.1% formic acid aqueous solution, and eluent B, which was methanol. During negative polarity mode, the elution solvent used was a mixture of eluent A (containing 5 mM ammonium acetate at pH 9.0) and eluent B (methanol). The elution process consisted of the following steps: 2% B for 1.5 min, 2–85% B for 3 min, 85–100% B for 10 min, 100–2% B for 10.1 min, and 2% B for 12 min. The Q ExactiveTM high-frequency mass spectrometer was used in both positive and negative polarity modes with a spray voltage of 3.5 kV, a capillary temperature of 320 °C, a sheath gas flow rate of 35 psi, an auxiliary gas flow rate of 10 L/min, an S-lens RF level of 60, and an auxiliary gas heater temperature of 350 °C [26].

#### 2.3.3. Data Analysis

After standardizing the raw peak area data to the total peak area, additional analysis was carried out. Principal component analysis was applied to evaluate the reproducibility of samples within the group and quality control samples. We classified the identified compounds and evaluated the pathway information using the Kyoto Encyclopedia of Genes and Genomes (KEGG), Human Metabolome Database, and LIPID MAPS structure database. Based on the *t*_test, we calculated the statistical significance (*p*-value) and the fold change (FC) of each metabolite between the two groups. The default criteria for selecting the differentially expressed metabolites were VIP (Variable Importance in Projection) > 1, *p*-value < 0.05, and FC (fold change) ≥ 2 or FC ≤ 0.5. The significance of KEGG pathway enrichment for different metabolites was determined by performing a statistical test called the hypergeometric distribution test.

### 2.4. Integrated 16S rRNA and Metagenomic Analyses

We performed correlation analysis using the Pearson correlation coefficient to identify the significant correlations between the differentially abundant bacterial genera obtained from the 16S rRNA analysis and the differentially abundant metabolites obtained from metabolomics analysis, followed by visualization using a chord diagram.

## 3. Results

### 3.1. Gut Microbial Taxonomy in Tibetan antelopes

**Figure 1:**
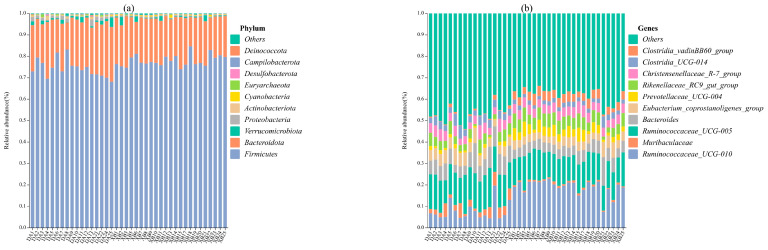
The relative abundance (top 10) of gut bacteria of *Tibetan antelopes* from different seasons at phylum (**a**) and genus (**b**) level. DA: warm season; XB: cold season.

The bacterial groups varied between the cold and warm seasons in *Tibetan antelopes*. At the phylum level (Figure 1a, Table S1), the intestinal microbe communities in the warm and cold seasons were dominated by *Firmicutes* (73.17  ±  4.03% vs. 78.05  ±  2.62%, respectively), *Bacteroidota* (22.73  ±  3.59% vs. 19.68  ±  2.54%, respectively), *Verrucomicrobiota* (1.31  ±  1.02% vs. 0.82  ±  1.01%, respectively), *Proteobacteria* (1.08  ±  0.79% vs. 0.36  ±  0.12%, respectively), and *Actinobacteriota* (0.52  ±  0.34% vs. 0.08  ±  0.02%, respectively), accounting for the majority (99%) of the detectable reads in all samples.

At the genus level (Figure 1b, Table S2), the primary bacterial genus found in the fecal samples of *Tibetan antelopes* during both the warm and cold seasons was *Ruminococcaceae UCG-005* (14.43  ±  2.10% vs. 12.97  ±  1.37%, respectively), followed by *Ruminococcaceae UCG-010* (7.75  ±  4.08% vs. 19.09  ±  3.48%, respectively), *Bacteroides* (7.64  ±  2.31% vs. 5.35  ±  1.89%, respectively), *Eubacterium coprostanoligenes group* (5.12  ±  1.14% vs. 4.14  ±  1.10%, respectively), and *Muribaculaceae* (4.39  ±  1.88% vs. 0.77  ±  0.21%, respectively).

### 3.2. Comparing the Microbiota Taxonomy between the Warm Seasons and Cold Seasons in Tibetan Antelopes

**Figure 2:**
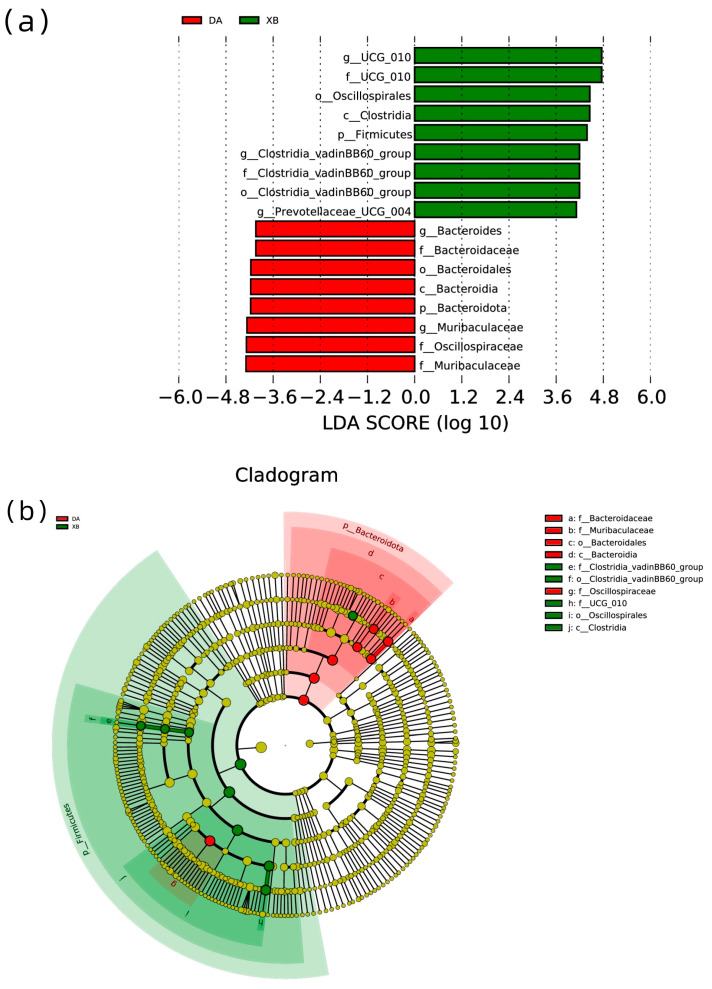
LEfSe analysis of Tibetan antelope intestinal bacterial biomarkers between two seasons. (**a**) The histogram of the LDA value distribution shows biomarker with significant statistical differences, and extent of histogram reflects degree of effect (LDA score). (**b**) The diameter of each circle reflects its richness. Multi-class analysis is flexible (at least one class difference). Inside-out circles reflect phylumto genus taxonomy. DA: warm season; XB: cold season.

The taxa were calculated to show significant differences between the cold and warm seasons at various levels by conducting linear discriminant analysis effect size analysis (Figure 2). At the phylum level, the relative abundance of *Bacteroidota* in the gut of the warm season group was significantly higher than in the cold season group. However, the relative abundance of *Firmicutes* was significantly enriched in the cold season group compared to the warm season group.

At the genus level, the relative abundance of *Muribaculaceae* and *Bacteroides* in the gut of the warm season group was remarkably higher than in the cold season group. However, the relative abundance of *Ruminococcaceae UCG-010*, *Prevotellaceae UCG-004*, and *Clostridia vadin BB60 group* was significantly enriched in the cold season group compared to the warm season group.

### 3.3. Gut Bacterial Community Diversity of Tibetan Antelopes

**Figure 3:**
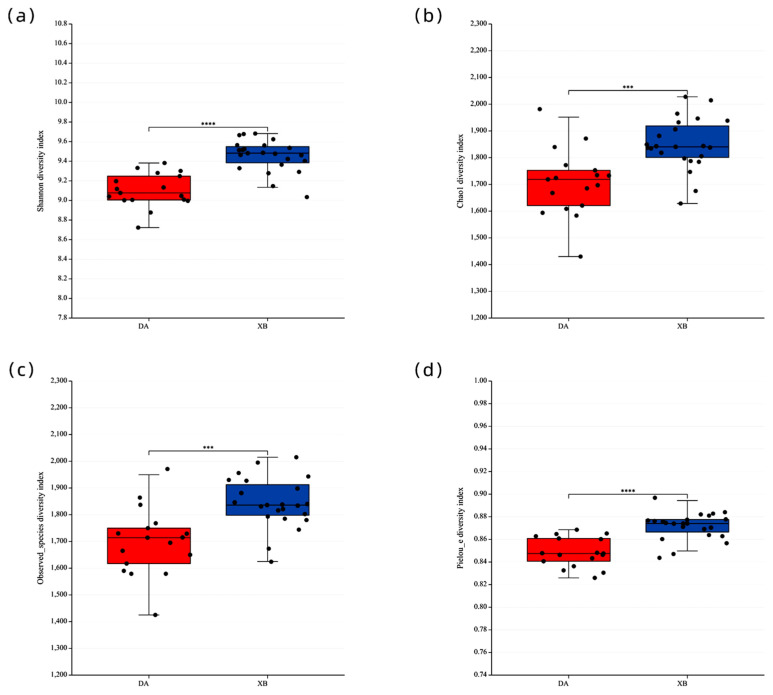

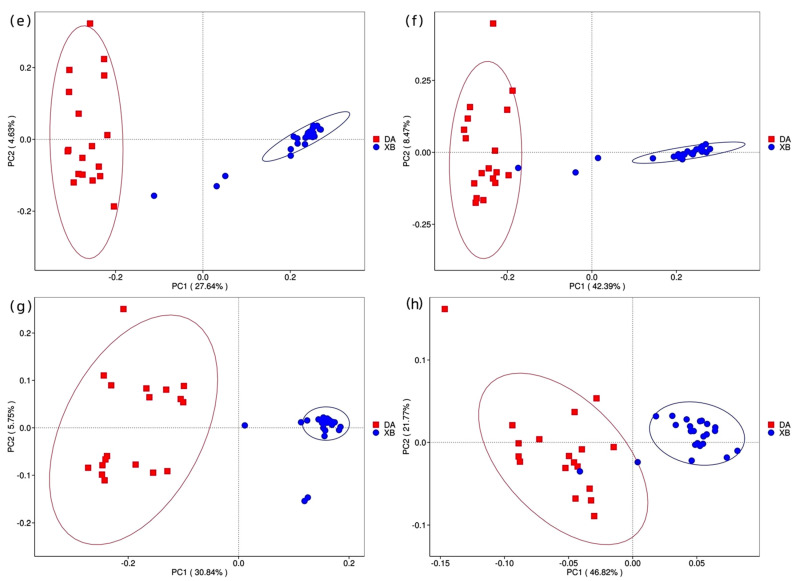
Alpha (**a**−**d**) and beta (**e**−**h**) diversity of the gut bacterial community of *Tibetan antelopes*. The alpha diversity of warm season and cold season *Tibetan antelopes* as measured by the Shannon (**a**), Chao1 (**b**), observed species (**c**), and pielou e (**d**). Principle co−ordinate analysis (PCoA) indicates separation by seasons based on Jaccard (**e**), Bray−Curtis (**f**), unweighted UniFrac (**g**) distances, and weighted UniFrac (**h**). DA: warm season; XB: cold season. *** *p*  <  0.001; **** *p*  <  0.0001.

The warm season group had a significantly lower level of diversity in the gut bacteria than the cold season group. (*p* ˂ 0.01, Figure 3a–d).

There were notable differences in the beta-diversity of *Tibetan antelopes* between the warm and cold seasons. A PCoA plot, using the Bray Curtis, Jaccard, Unweighted Unifrac, and Weighted Unifrac distance matrix, clearly showed a distinct separation of the fecal microbiota between the *Tibetan antelopes* in the warm and cold seasons (Figure 3e–h).

### 3.4. Predicted Microbiota Functional Categories

**Figure 4:**
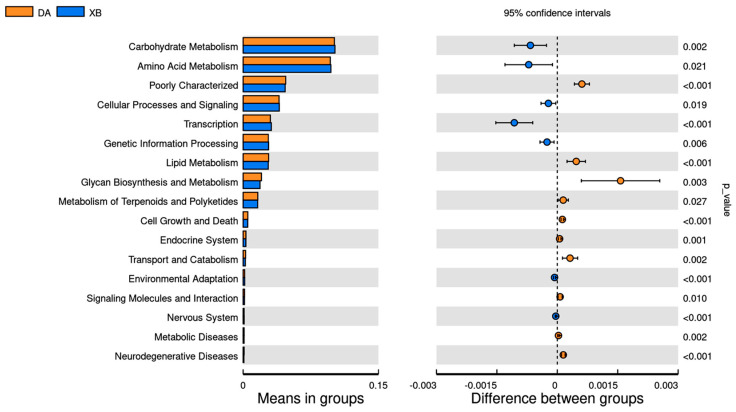
The predicted gut bacterial functional categories and pathways of *Tibetan antelopes* from two seasons. The left figure displays the functional abundance differences between groups, where each bar represents the mean abundance of significantly different functions in each group. The right figure shows the confidence intervals of the inter-group differences, where the leftmost point of each circle represents the lower limit of the 95% confidence interval for the mean difference, and the rightmost point represents the upper limit. The center of each circle represents the difference in means, and the color of the circle represents the group with a higher mean. The significance test *p*-value for the corresponding differentially abundant function is presented at the far right of the display. DA: warm season; XB: cold season.

The analysis of functional predictions in the KEGG database revealed that the gut microbiota of *Tibetan antelopes* primarily play a role in carbohydrate metabolism, amino acid metabolism, cellular processes and signaling, transcription, and genetic information processing. The KEGG database also indicated that the two major metabolic functions of *Tibetan antelopes* exhibited seasonal variations. In general, these functions were higher in the cold season than in the warm season (*p* < 0.05, Figure 4, Table S3).

### 3.5. Season-Associated Changes in the Fecal Metabolome

#### 3.5.1. Multivariate Statistical Analysis

**Figure 5:**
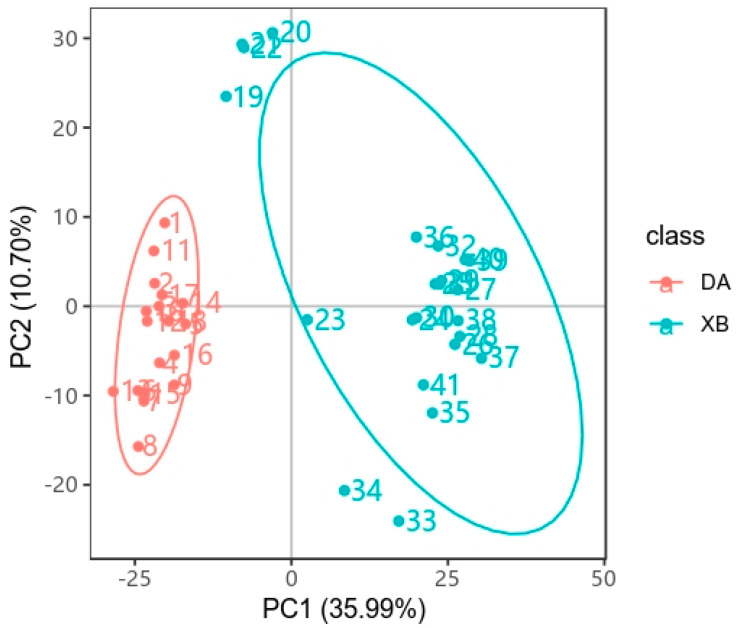
Principal component analysis. DA: warm season; XB: cold season.

The results from the principal component analysis indicated a distinction in the metabolite profiles between the warm and cold seasons in *Tibetan antelopes* (Figure 5).

#### 3.5.2. Differential Metabolite Analysis

**Figure 6:**
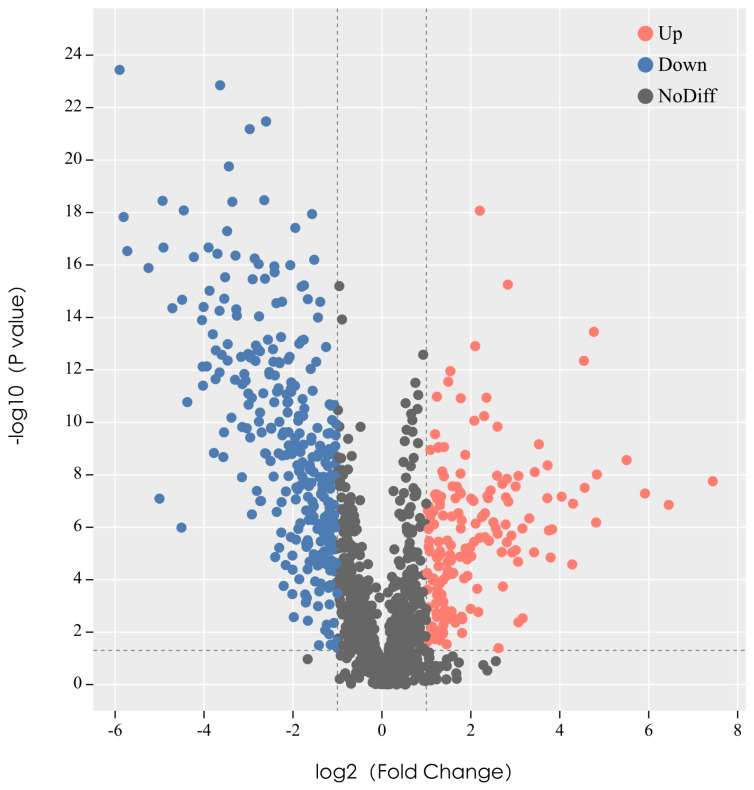
Volcano map of differential metabolites. Each point in the volcanic map represents a metabolite, the abscissa represents the changes in each substance compared between the groups, and the ordinate represents the *p*-value of the *t*-test.

In the XB and DA groups, a total of 1307 metabolites were annotated. Out of these, 532 metabolites were found to have differential expression (Tables S4 and S5). The outcomes of the screened differential metabolites are displayed in the format of a volcano plot in all ion modes (Figure 6). In the XB group, there were a total of 171 metabolites that showed increased levels, while 361 metabolites showed decreased levels compared to the DA group. The top 10 upregulated metabolites were 5-S-Methyl-5′-thioadenosine, Cyclohexyl fentanyl-d5, isoleucine, Psychosine, 14,15-Leukotriene E4, gamma-Glutamylcysteine, 3-(4-chloropheny)-5-methyl-2,5-dihydro-1,24-0xadiazole, and Lysops 22:6. The top 10 downregulated metabolites were 2-Deoxyinosine, 3-Hydroxystanozololand asiaticoside B, 6-methoxy-2-phenyl-3,4-dihydro-2H-1-benzopyran-4-one, Deoxyguanosine, Maltotriose, DGDG O-8:0-2:0, Hydrocortisone acetate, N-Acetyl-Asp-Glu, Guanine, and Gedunin.

**Figure 7:**
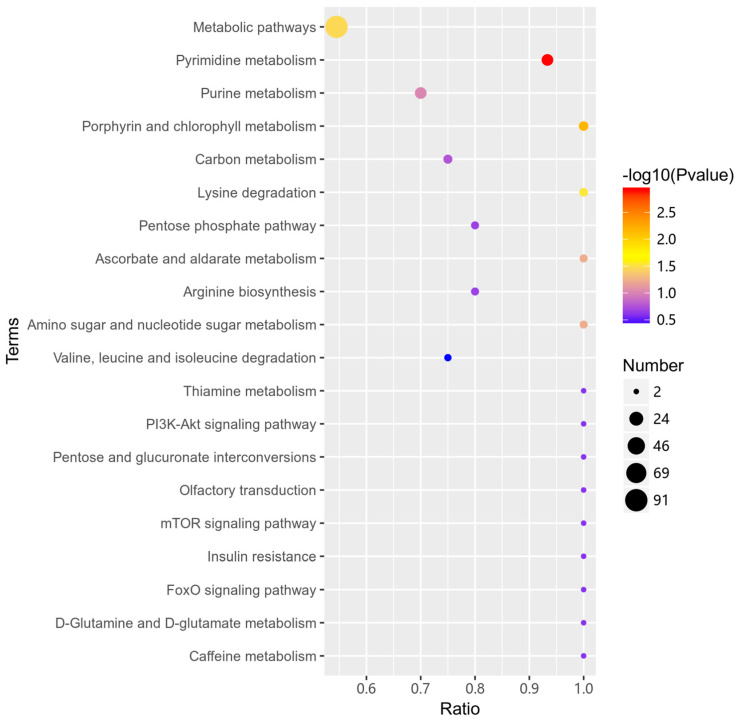
Bubble plot of KEGG enrichment. The size of the dots represents the number of differential metabolites in the corresponding pathway. The larger the dot is, the more differential metabolites there are in that pathway.

A total of 532 differential metabolites were annotated using the KEGG database. A total of 62 differential metabolic pathways were identified (Table S6). The top 20 pathways were mainly enriched in pyrimidine metabolism, purine metabolism, porphyrin and chlorophyll metabolism, carbon metabolism, and lysine degradation. Out of all the pathways, the pyrimidine metabolism had the largest number of annotated metabolites, including Deoxycytidine, Cytidine-5′-monophosphate, 5-Methylcytosine, Thymine, Pseudouridine, dCDP, dUMP, Uracil, UDP, Cytosine, 2-Deoxyuridine, UMP, Uridine, and Thymidine (Figure 7).

### 3.6. Integrated Analysis of 16S rRNA and Metabolomic

**Figure 8:**
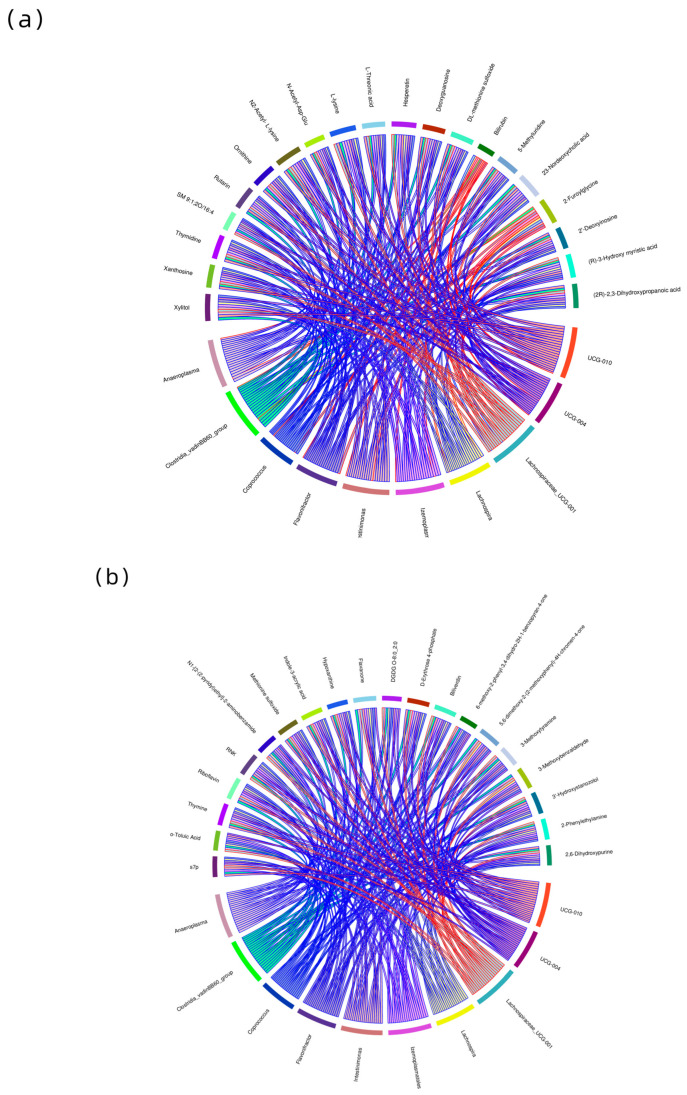
Chord diagram of correlation ((**a**) is the negative ion mode; (**b**) is the positive ion mode). Each node represents a differential bacterial genus and differential metabolites. The width of the strings represents the strength of the correlation. The color of the string border represents the correlation, with red indicating positive correlation and blue indicating negative correlation.

Statistical analysis was performed on the gut microbiota and metabolites that showed significant differences in abundance (Tables S7 and S8). For instance, *Anaeroplasma* was positively associated with 2-Furoyiglycine but negatively associated with L-lysine and Hesperetin. *Ruminococcaceae UCG-010* was negatively correlated with N2-Acetyl- L-lysine and DL-methionine sulfoxide. *Lachnospiraceae UCG-001* was positively correlated with 2,6-Dihydroxypurine, 2-Phenylethylamine, 3-Hydroxystanozolol, 3-Methoxybenzaldehyde, Biliverdin, Flavanone, Hypoxanthine, Methionine sulfoxide, Indole-3-acrylic acid, and Thymine. Flavonifractor was negatively correlated with Hypoxanthine, Riboflavin, and Thymine (Figure 8).

## 4. Discussion

### 4.1. Composition of the Gut Bacterial Community

The gut microbiota have a significant impact on the host’s metabolism, immune system, and overall internal balance [27,28,29]. Studying the classification and functional differences of gut microbiota related to seasonal adaptation can contribute to a deeper understanding of the mechanisms behind animal adaptation to seasonal changes. The dominant bacterial species and their relative abundance in the gut bacterial community of *Tibetan antelopes* vary significantly between the warm and cold seasons. This difference between the seasons has been previously observed in other research studies [30,31]. *Firmicutes* and *Bacteroidetes* are the dominant core bacteria in *Tibetan antelopes*, with the highest relative abundance. This bacterial community composition was consistent with that of other Bovidaes such as Przewalski’s Gazelle [32] and the Tibetan Gazelle [33]. *Firmicutes* are highly prevalent in the gastrointestinal tract of Bovidaes and consist of numerous groups that are involved in cellulose metabolism, including *Ruminococcus* and *Clostridia* [33]. *Firmicutes* play a vital role in the gastrointestinal system of herbivorous animals, as they are primarily responsible for cellulose decomposition and conversion into volatile fatty acids. This process aids in the effective breakdown of food and promotes the host’s growth and development. The enrichment of *Firmicutes* significantly contributes to the ability of *Tibetan antelopes* to acquire ample nutrients from their diet, while also influencing the metabolic function of the fecal microbiota. *Bacteroidetes*, on the other hand, plays a crucial role in boosting organism metabolism, supporting the growth of the gastrointestinal immune system, and actively engaging in the metabolisms of bile acids, proteins, and fats. Furthermore, it possesses a regulatory impact on the process of carbohydrate metabolism and can generate specific glycans and polysaccharides known for their potent anti-inflammatory properties [34]. The proportions of *Firmicutes* and *Bacteroidetes* varied significantly throughout the seasons in *Tibetan antelopes*. In the cold season, the availability of food is scarce. *Tibetan antelopes* consume a higher proportion of high-fiber foods during the cold season than during the warm season. Consequently, a higher abundance of Firmicutes can assist *Tibetan antelopes* in better digesting and absorbing these high-fiber foods. This is beneficial for *Tibetan antelopes* in adapting to the cold season.

### 4.2. Structure and Diversity of the Gut Bacterial Community

Principal co-ordinate analysis revealed that the structure and composition of the gut microbiota in *Tibetan antelopes* varied across different seasons, similar to previous research findings [10]. This variation is likely due to the consumption of different types of food. Specifically, during the cold season, *Tibetan antelopes* had a significantly greater proportion of Gramineae in their diet compared to other seasons. Our findings indicated that the alpha-diversity of the gut microbiota in *Tibetan antelopes* was greater during the cold season than during the warm season. Alpha-diversity alterations may be attributed to environmental fluctuations. Previous studies have demonstrated that increased alpha-diversity promotes a stable and intricate intestinal micro-organism composition. This enhances the resistance to external disruptions and improves adaptability, ultimately benefiting the overall health of the host [35]. As a result, increased alpha-diversity in the gut microbiota of *Tibetan antelopes* in cold seasons can improve the resistance to harsh environmental factors, reduce the harsh environmental effects, and promote the uptake and use of fiber-rich foods and nutrients.

From the perspective of the functions of gut microbiota, many pathways of metabolism and synthesis are remarkably upregulated. This indicates that *Tibetan antelopes* have higher nutritional requirements in the cold season, which, accordingly, enhances the metabolism and synthesis of amino acids and carbohydrates. This adaptation is advantageous for *Tibetan antelopes* to survive and thrive in the harsh winter environment.

### 4.3. Intestinal Metabolomics Response to Seasonal Changes

In the present study, the differential metabolites between cold season and warm season *Tibetan antelopes* were compared utilizing untargeted metabolomics. PCA effectively differentiated the metabolite spectra between the cold season and warm season groups. Interestingly, the differential metabolites were mainly enriched in pyrimidine metabolism and purine metabolism.

Nucleotide metabolism is crucial for various biological processes such as synthesizing genetic materials, providing energy, regulating metabolism, acting as messenger molecules, and forming coenzymes, including purine metabolism and pyrimidine metabolism [36,37]. Therefore, the synthesis and degradation pathways of pyrimidine and purine in organisms are particularly important for their normal functioning. This research reveals that 28 different metabolites enriched in purine metabolism and pyrimidine metabolism pathways are found in high abundance in the feces of *Tibetan antelopes* during the warm season. This suggests that, during the warm season, *Tibetan antelopes* have a high capacity for purine metabolism and pyrimidine metabolism. This could be due to the intense ultraviolet radiation in the high-altitude region where *Tibetan antelopes* reside during the warm seasons. Ultraviolet (UV) irradiation causes DNA damage [38,39], and *Tibetan antelopes* in the warm seasons may repair UV-damaged DNA through higher levels of pyrimidine and purine metabolism to adapt to the intense UV radiation in the high-altitude environment.

### 4.4. Integration of Metagenomic and 16S rRNA

The integrated analysis of the gut microbiota and metabolome is beneficial for understanding the connection between gut bacteria and metabolites. Seasonal variation altered the gut microbiota composition and metabolism of *Tibetan antelopes*, which also had effects on each other, ultimately impacting their metabolism, growth, immunity, and other physiological aspects.

In this study, there was a significant correlation between the differential microbiota and the differential metabolites. Among them, the differential microbiota that were most significantly correlated with differential metabolites were *Ruminococcaceae UCG-010*, *Clostridia vadinBB60 group,* and *Lachnospiraceae UCG-001*. According to research, *Ruminococcaceae UCG-010* is connected to the breakdown of starch and fiber in ruminants [40]. The *Clostridia vadinBB60 group* is related to the higher fat deposition [41]. *Lachnospiraceae UCG-001* is present in the intestines of most healthy individuals and may be a potential probiotic. It plays a role in the metabolism of various carbohydrates, particularly in the strong ability to metabolize pectin found in fruits and vegetables. It also produces acetic acid and butyric acid through fermentation, providing energy for the host [42,43]. The relative abundance of *Ruminococcaceae UCG-010* and the *Clostridia vadinBB60 group* in the gut of the cold season group was remarkably higher than in the warm season group. Thus, we speculate that *Ruminococcaceae UCG-010*, the *Clostridia vadinBB60 group* and *Lachnospiraceae UCG-001* might improve *Tibetan antelopes*’ adaptability to seasonal variation.

## 5. Conclusions

The research examined the variations in the metabolites and gut microbiota across different seasons by using untargeted metabolomics and 16S rRNA sequencing of fecal samples from *Tibetan antelopes* in different seasons. The analysis revealed noticeable seasonal variations in metabolites and the composition and functionality of the gut microbiota. Thus, investigating the gut microbiota and metabolites of *Tibetan antelopes* can yield crucial insights into their overall health, welfare, and the threats they face in their natural habitat. This knowledge can be utilized to aid targeted conservation efforts aimed at promoting the reproduction and long-term survival of *Tibetan antelopes*.

## Data Availability

The data presented in the study are deposited in the NCBI GenBank, accession number PRJNA1011819 (https://www.ncbi.nlm.nih.gov/bioproject/PRJNA1011819 (accessed on 1 September 2023)).

## Supplementary Materials

The following supporting information can be downloaded at: https://www.mdpi.com/article/10.3390/ani13223569/s1, Table S1: phylum relative abundance; Table S2: genus relative abundance; Table S3: function relative abundance; Table S4: neg_differential metabolites; Table S5: pos_differential metabolites; Table S6: 62 differential metabolic pathways; Table S7: neg_ Integrated analysis of 16S rRNA and metabolomic; Table S8: pos_ Integrated analysis of 16S rRNA and metabolomic.
